# Differential Impacts of Land-Based Sources of Pollution on the Microbiota of Southeast Florida Coral Reefs

**DOI:** 10.1128/AEM.03378-16

**Published:** 2017-05-01

**Authors:** Christopher Staley, Thomas Kaiser, Maribeth L. Gidley, Ian C. Enochs, Paul R. Jones, Kelly D. Goodwin, Christopher D. Sinigalliano, Michael J. Sadowsky, Chan Lan Chun

**Affiliations:** a BioTechnology Institute, University of Minnesota, St. Paul, Minnesota, USA; b Ocean Chemistry and Ecosystems Division, Atlantic Oceanographic and Meteorological Laboratory, National Oceanic and Atmospheric Administration, Miami, Florida, USA; c Cooperative Institute for Marine and Atmospheric Studies, University of Miami, Miami, Florida, USA; d Department of Civil Engineering and National Resources Research Institute, University of Minnesota Duluth, Duluth, Minnesota, USA; Georgia Institute of Technology

**Keywords:** coral, land-based sources of pollution, microbial ecology, microbial source tracking, next-generation sequencing

## Abstract

Coral reefs are dynamic ecosystems known for decades to be endangered due, in large part, to anthropogenic impacts from land-based sources of pollution (LBSP). In this study, we utilized an Illumina-based next-generation sequencing approach to characterize prokaryotic and fungal communities from samples collected off the southeast coast of Florida. Water samples from coastal inlet discharges, oceanic outfalls of municipal wastewater treatment plants, treated wastewater effluent before discharge, open ocean samples, and coral tissue samples (mucus and polyps) were characterized to determine the relationships between microbial communities in these matrices and those in reef water and coral tissues. Significant differences in microbial communities were noted among all sample types but varied between sampling areas. Contamination from outfalls was found to be the greatest potential source of LBSP influencing native microbial community structure among all reef samples, although pollution from inlets was also noted. Notably, reef water and coral tissue communities were found to be more greatly impacted by LBSP at southern reefs, which also experienced the most degradation during the course of the study. The results of this study provide new insights into how microbial communities from LBSP can impact coral reefs in southeast Florida and suggest that wastewater outfalls may have a greater influence on the microbial diversity and structure of these reef communities than do contaminants carried in runoff, although the influences of runoff and coastal inlet discharge on coral reefs are still substantial.

**IMPORTANCE** Coral reefs are known to be endangered due to sewage discharge and to runoff of nutrients, pesticides, and other substances associated with anthropogenic activity. Here, we used next-generation sequencing to characterize the microbial communities of potential contaminant sources in order to determine how environmental discharges of microbiota and their genetic material may influence the microbiomes of coral reef communities and coastal receiving waters. Runoff delivered through inlet discharges impacted coral microbial communities, but impacts from oceanic outfalls carrying treated wastewater were greater. Geographic differences in the degree of impact suggest that coral microbiomes may be influenced by the microbiological quality of treated wastewater.

## INTRODUCTION

Coral reefs are highly diverse ecosystems that play crucial roles in maintaining marine biodiversity and productivity and coastal protection, and they serve as a source of food and recreation (1). Corals exist as holobionts (2) composed of a coral polyp, endosymbiotic zooxanthellae (Symbiodinium spp.) (3), bacteria (4), fungi (5), archaea (6), and viruses (7). The interactions of all of the constituents of the coral microbiome have recently been described as dynamic, changing in response to seasonal variations and with disease state (8). Due to global climate change and other anthropogenic impacts on the coral microbiome (1, 9), coral reefs have been recognized as endangered ecosystems for the last several decades (10). Estimates are that 20% of coral reefs globally are already lost, while approximately 24% face imminent risk, and another 26% may be facing severe damage (1).

A core microbiome among corals is defined functionally rather than based on the presence of specific taxa (4, 11–13), similar to what is found in humans (14). The coral microbiome has recently been implicated in the onset of reef diseases, where stresses on the microbiome (e.g., elevated temperature) disturb normal host resistance and/or restriction from other members of the microbiome. Consequently, this allows overgrowth of typically commensal taxa and various opportunistic pathogens (11, 15). Variation in the coral microbiome has also been shown to follow seasonal dynamics (8, 12, 16), with temperature having a more significant impact on community composition than the disease state (8, 12). However, the functional states of the coral microbiome show some plasticity, adapting to geographical differences and nutrient availability (13).

Anthropogenic impacts, primarily in the form of terrestrial runoff, also contribute to nutrient loading, sediment deposition, and the transport of pesticides, pharmaceuticals, and other harmful chemicals to nearby coral reefs, stressing coral communities (17, 18). The parameters influenced by these land-based sources of pollution (LBSP) have been shown to be dependent on the surrounding land cover (19). Not surprisingly, the concentration of pollutants and the extent of discharge from LBSP are directly related to rain events that increase river flows (16, 20, 21). Importantly, LBSP and their associated changes in water chemistry also influence proximate marine communities (16, 22). During periods of high flow (i.e., during rain events), the abundances of Burkholderiales and Sphingobacteriales, common to river samples, increased in marine waters and were correlated with changes in physicochemical parameters (16).

It was previously suggested that threats to corals can be broadly divided into global threats, representing climatic or ecological phenomena that are difficult for resource managers to control, and local threats, which refer to specific anthropogenic activities that can be directly modulated and regulated (1). Probiotic approaches, such as phage therapy, bioaugmentation, or adaptation of the commensal coral microbiota, have been suggested as means to combat more global threats (11, 23, 24), but the ethics and potential consequences of these actions must be carefully considered. Locally, nutrient runoff, sewage discharge, coastal construction, and overfishing represent more manageable stressors to protect coral communities (reviewed in reference 1). While the negative consequences of these actions have been generally understood for decades, their impacts on the coral microbiome have only recently received attention (16, 22).

In this study, we used a next-generation sequencing approach to characterize the microbial communities from LBSP (coastal inlets, oceanic outfalls from wastewater treatment plants, and wastewater treatment effluent), coral reef waters, and coral tissues (mucus and polyps) among coral reefs off the southeastern coast of Florida, offshore of the Miami-Dade and Broward counties. We hypothesized that the impacts of various LBSP on microbial (prokaryotic and fungal) communities in nearby coral reefs and tissue samples would vary as a result of demographic and hydrological differences associated with sampling sites, and that the relative degree of source impacts could be determined based on exchange between microbial communities from LBSP and reef waters or coral tissues. Here, potential contributions from LBSP were evaluated using SourceTracker analysis (25–27), which utilizes a Bayesian approach to assign operational taxonomic units (OTUs) from sink communities to sources. This work is a component of a larger Florida Area Coastal Environment (FACE) program study (http://www.aoml.noaa.gov/themes/CoastalRegional/projects/FACE/faceweb.htm). The goals of this program are to investigate nutrient concentration and transport from LBSPs, perform coral benthic surveys of coral cover and health, and monitor microbiological water quality of the southeast Florida sentinel coral reef sites used in this study. The results of these other source tracking investigations from the broader FACE study are primarily reported elsewhere (28, 29), and the work we report here focuses on the characterization of microbial communities and potential sources of contamination using next-generation sequencing.

## RESULTS

### Monitoring nutrients and microbiological qualities of water.

Water samples (i.e., inlet, reef water, open ocean water, and outfall samples) differed significantly in all physiochemical parameters measured (Table 1), except for temperature (*P* = 0.114) and dissolved oxygen (*P* = 0.157), when grouped by sample type. Inlet samples had greater colored dissolved organic matter, turbidity, nitrate plus nitrite N, and chlorophyll *a* concentrations and lower salinity than all other sample types (*P* < 0.0001 for all parameters). Outfall samples had significantly greater concentrations of nitrogen, total nitrogen, and total phosphorus (*P* < 0.0001). Water density also varied significantly among sample types (*P* = 0.001), with reef water and outfall samples having intermediate densities between those observed for open ocean and inlet samples.

**TABLE 1:** Physicochemical parameters measured among water samples^*a*^

Parameter	Value for sample type			
	Open ocean	Inlet	Reef water	Outfall
*n*	27	25	157	55
Salinity (‰)	36.2 ± 0.2	35.1 ± 1.5	35.9 ± 0.9	35.7 ± 1.0
Temp (°C)	25.7 ± 2.0	27.1 ± 2.7	26.5 ± 2.2	26.8 ± 2.1
Density (kg · m^−3^)	24.0 ± 0.6	22.7 ± 1.5	23.5 ± 1.2	23.3 ± 1.2
CDOM (µg · liter^−1^)	1.1 ± 0.8	11.0 ± 11.5	1.6 ± 3.2	2.2 ± 2.2
Turbidity (NTU)	1.3 ± 0.9	4.1 ± 2.4	1.4 ± 1.5	1.2 ± 0.6
DO (mg · liter^−1^)	6.6 ± 0.2	6.4 ± 0.3	6.5 ± 0.3	6.5 ± 0.3
N+N (µM)	0.3 ± 0.0	1.0 ± 0.9	0.4 ± 0.3	0.5 ± 0.5
TKN (µM)	3.9 ± 1.8	6.6 ± 3.5	4.1 ± 1.8	11.2 ± 16.2
TN (µM)	4.2 ± 1.8	7.6 ± 3.9	4.5 ± 1.8	11.7 ± 16.3
TP (µM)	0.3 ± 0.1	0.3 ± 0.1	0.2 ± 0.1	0.5 ± 0.5
Chl-a (µg · liter^−1^)	0.4 ± 0.2	1.0 ± 0.5	0.4 ± 0.3	0.4 ± 0.2

a Values are means ± standard deviations among all samples. Parameters measured include salinity, temperature, density, colored dissolved organic matter (CDOM), turbidity, dissolved oxygen (DO), nitrate + nitrite (N+N), total Kjeldahl nitrogen (TKN), total nitrogen (TN), total phosphorus (TP), and chlorophyll *a* (Chl-a).

As reported previously (29), pepper mild mottle virus (PMMoV) and human polyomavirus (HPyV) were quantified at inlet and outfall sites in 2014 (see Table S1 in the supplemental material). Due to the low frequency of quantifiable results, HPyV data were treated as binary (presence/absence) data. Pepper mild mottle viruses were generally on the order of 10^4^ gene copies · liter^−1^ at outfalls and 10^2^ gene copies · liter^−1^ at inlet sites. Human polyomavirus was similarly detected more frequently at outfalls, with nearly two-thirds of the samples positive at the Miami Central outfall, compared to only about one-third (38.9%) at the Miami North outfall, with 16.7 to 33.3% of inlet samples testing positive for HPyV.

### Alpha diversity of prokaryotic community.

Sequence analysis found a range of 119 to 3,943 OTUs among all samples, with a mean ± standard deviation Good's coverage of 98.8% ± 0.8%. When measured by the Shannon index, samples collected from inlets had significantly lower alpha diversity (Table 2) than did the ocean and reef sites, while the outfall and wastewater treatment plant (WWTP) effluent samples had intermediate Shannon diversity values. Prokaryotic communities associated with corals (mucus and polyps) had significantly lower diversity than did all water samples. However, no significant differences (*P* > 0.05) were observed in abundance-based coverage estimate (ACE) richness among sample types. Within a given sample type, differences in alpha diversity did not differ significantly by site, except among coral tissues, where ACE richness tended to increase at reef sites as follows: Barracuda = Emerald < Oakland Ridge < Pillars (*P* = 0.034, Tukey's *post hoc P* ≥ 0.093).

**TABLE 2:** Coverage and alpha diversity indices for bacterial communities among all samples collected, consolidated by sample type^*a*^

Sample type	*n*	Coverage (%)	S_obs_^*b*^	Index^*c*^	
				Shannon	ACE
Open ocean	25	98.7 ± 0.4	1,095 ± 208	4.42 ± 0.22 A	2,243 ± 804
Inlet	27	98.9 ± 0.7	765 ± 396	3.57 ± 0.67 B,C	2,070 ± 1,386
Reef water	147	98.8 ± 0.6	968 ± 395	4.19 ± 0.60 A	2,001 ± 1,144
Outfall	53	99.0 ± 0.7	875 ± 541	3.99 ± 0.79 A,B	1,774 ± 1,180
WWTP	9	99.0 ± 0.9	821 ± 643	3.66 ± 1.10 A,B,C	1,765 ± 1,569
Coral tissue	87	98.7 ± 1.1	1,184 ± 838	3.31 ± 1.39 C	1,999 ± 1,629

a Values are means ± standard deviations among all samples.

b S_obs_, number of OTUs observed.

c For Shannon index, sample groups sharing the same letter did not differ significantly in alpha diversity by Tukey's *post hoc* test (*P* > 0.05). Overall *P* values for Shannon's index and ACE index are <0.0001 and 0.734, respectively.

### Prokaryotic community composition.

**FIG 1:**
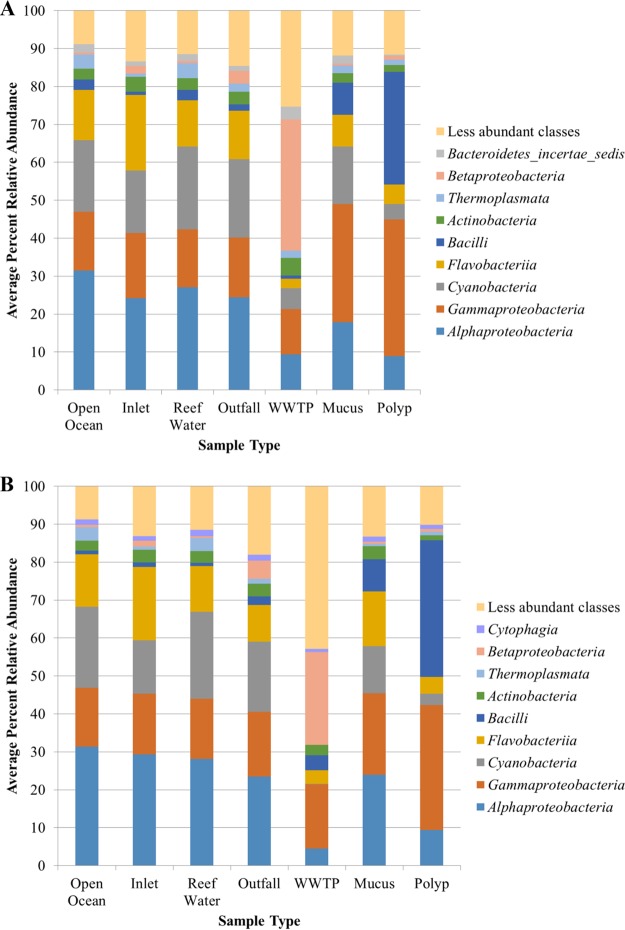
Distribution of abundant bacterial classes among sample types collected from the northern (A) and southern (B) cruise regions.

Prokaryotic community composition was similar among all environmental water sample types (reef, open ocean water, inlet, and outfall water), with high relative abundances of Alphaproteobacteria and Cyanobacteria (Fig. 1). In contrast, the WWTP effluent samples, before oceanic discharge, were predominantly composed of Betaproteobacteria. Coral mucus and polyp samples had greater relative abundances of Gammaproteobacteria, with a greater relative abundance of Bacilli among polyps than that with mucus samples. At higher taxonomic resolution, coral tissue communities primarily consisted of Endozoicomonas and Bacillus, with a relatively greater percentage of the community that could not be assigned to the genus level (Fig. S1). The percentage of Endozoicomonas was significantly different among tissue samples from each reef (*P* = 0.038), and tended to be higher at the Oakland Ridge reef. The abundances of Bacillus and Paenibacillus also differed significantly (*P* = 0.008 and 0.004, respectively) and tended to be greater among polyps collected from Barracuda and Emerald reefs. Among all tissue samples, the percentage of unclassified reads was lower for polyp samples (*P* < 0.0001).

### Beta diversity of prokaryotic communities.

The prokaryotic composition, as evaluated by analysis of similarity (ANOSIM), did not differ significantly between open ocean and reef water sample types when grouped by northern or southern sampling areas (*P* = 0.911 to 0.914). Prokaryotic communities in inlet and WWTP samples generally differed significantly from every other sample type (*P* ≤ 0.024 and ≤0.005, respectively), except for the inlet and coral mucus samples collected from the southern area, which did not differ significantly (*P* = 0.329). Community composition of open ocean sites also did not differ from outfall samples in the northern region (*P* = 0.104), but community composition was significantly (*P* = 0.015) different among sampling sites in the southern sampling area. Reef communities differed from those of outfalls in either region (*P* ≤ 0.002). Generally, the prokaryotic communities in coral mucus and polyp samples were significantly different from all the other sample types (*P* ≤ 0.009), except as noted above. Communities from mucus and polyps did not differ from each other in the northern sampling region (*P* = 0.225), but they were significantly different among samples collected in the southern region (*P* = 0.001). Samples from different sites of the same sample type did not harbor significantly different prokaryotic communities (*P* ≥ 0.119).

**FIG 2:**
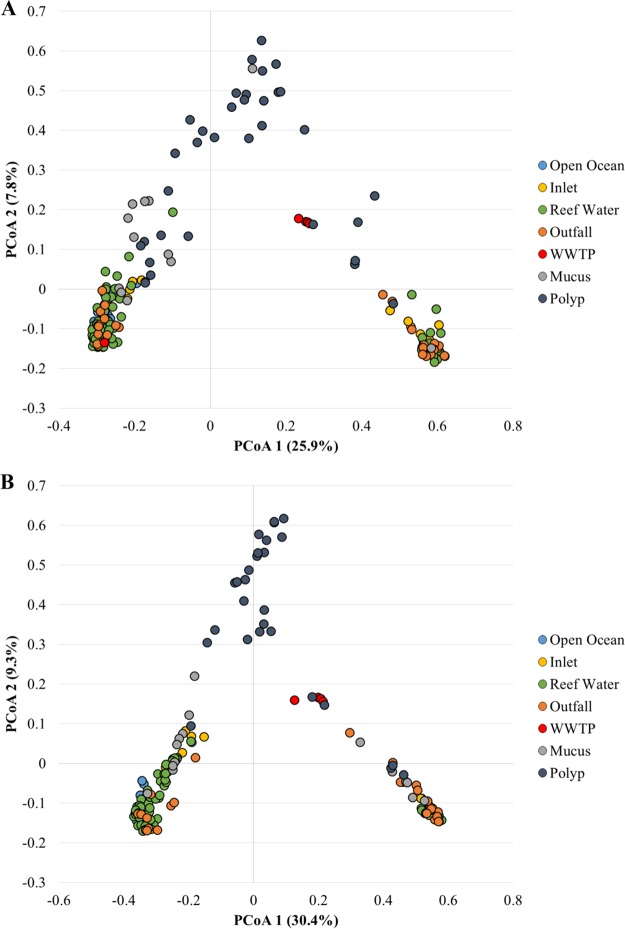
Principal-coordinate analyses of bacterial communities collected from the northern (*r*^2^ = 0.818) (A) and southern (*r*^2^ = 0.787) (B) sampling regions.

Ordination of Bray-Curtis distances by principal-coordinate analyses (PCoA; Fig. 2) revealed clustering by sample type, and this separation was supported by analysis of molecular variance (AMOVA) (*P* < 0.001) for both sampling areas. Water samples tended to cluster together, apart from coral tissue and WWTP effluent samples. Similar to the ANOSIM results, in the southern region, *post hoc* tests revealed no significant separation of inlet from coral mucus samples (*P* = 0.225). Differences in sample type were primarily attributable to the families Cyanobacteria group IIa (GpIIa), Flavobacteriaceae, “Candidatus Pelagibacter,” Rhodospirillaceae, and Rhodobacteraceae among water samples, and these families were less abundant in outfall samples (Fig. S1). The WWTP samples showed taxonomic variability between sampling areas, with the WWTP samples associated with the northern region (Miami North) primarily composed of Burkholderiaceae, while those associated with the southern region (Miami Central) were predominantly composed of a large number of less abundant families. Coral tissue samples had significantly greater abundances of Bacillaceae and Hahellaceae.

Redundancy analysis (RDA) generally supported the results of the Kruskal-Wallis test (Fig. S2 and S3). Coral tissue samples were more strongly associated with increased abundances of Bacillaceae and Hahellaceae, while inlet samples were associated with greater abundances of Flavobacteriaceae and Rhodobacteraceae. Abundances of Cyanobacteria group II were also associated with reef water samples. Little separation was observed between open ocean and outfall samples, which clustered with ubiquitous marine families. Of note, water samples from the Pillars reef site were more closely related to inlet samples than were samples from other reefs.

### Fungal community diversity and composition.

**FIG 3:**
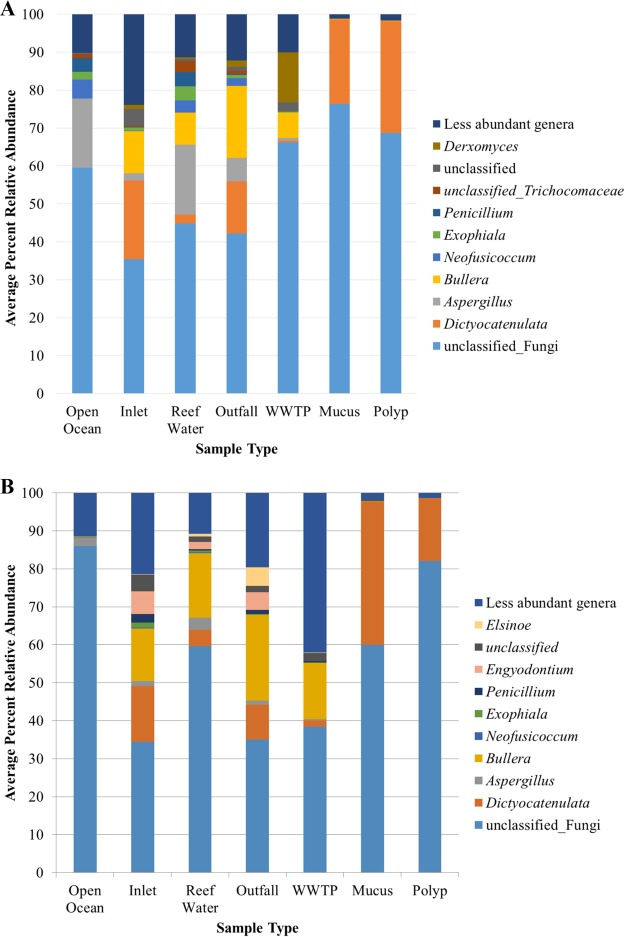
Distribution of fungal genera in samples collected from the northern (A) and southern (B) sampling regions.

Fungal community coverage was estimated at 97.9% ± 1.5% among all samples, with between 62 and 1,125 OTUs in individual samples. Differences in alpha diversity among fungal communities (Table 3) generally corresponded to those observed for prokaryotic communities and are described in detail in the supplemental results. A large portion of the fungal community among all samples could not be assigned to a phylum (Fig. 3). Among the genera that could be classified, Dictyocatenulata and Bullera spp. were predominantly found in water and WWTP samples, while Dictyocatenulata was among the only genus that could be classified from coral tissue samples. Aspergillus was also somewhat abundant among samples collected in the northern region, especially in ocean and reef water samples.

**TABLE 3:** Coverage and alpha diversity indices for fungal communities among all samples collected, consolidated by sample type^*a*^

Sample type	*n*	Coverage (%)	S_obs_^*b*^	Index^*c*^	
				Shannon	ACE
Open ocean	17	97.1 ± 1.1	614 ± 238	3.74 ± 1.08 A,B	1457 ± 560 A
Inlet	24	96.6 ± 1.0	630 ± 184	3.27 ± 0.95 B	2,012 ± 759 B,C
Reef water	110	97.2 ± 1.1	558 ± 212	3.33 ± 1.02 B	1,536 ± 624 A
Outfall	45	97.1 ± 1.1	540 ± 208	3.06 ± 1.10 B	1,691 ± 665 A,B
WWTP	10	96.0 ± 0.6	743 ± 125	4.33 ± 0.54 A	2,376 ± 566 C
Coral tissue	117	99.5 ± 0.2	106 ± 27	1.26 ± 0.55 C	352 ± 180 D

a Values are means ± standard deviations among all samples.

b S_obs_, number of OTUs observed.

c Sample groups sharing the same letter did not differ significantly in alpha diversity by Tukey's *post hoc* test (*P* > 0.05). Overall *P* values for Shannon's index and ACE index are both <0.0001.

Differences in beta diversity among fungal communities from LBSP (i.e., inlets and outfalls), reef water, and open ocean samples were similar to those described for prokaryotic communities (described in detail in the supplemental results). In both northern and southern sampling areas, coral mucus and polyp communities differed significantly in community composition from those of other sample types (*P* ≤ 0.006 and ≤0.040, with respect to sampling area), but communities characterized from mucus and polyps were not significantly different (*P* = 0.478 and 0.091, respectively). In both sampling regions, no differences in beta diversity were observed among samples of the same type between sampling sites (*P* ≥ 0.113). Ordination of samples by PCoA showed poor correlations to distance matrices (*r*^2^ ≤ 0.113) and did not show any trends in sample clustering (data not shown).

### Influence of land-based sources of pollution.

**FIG 4:**
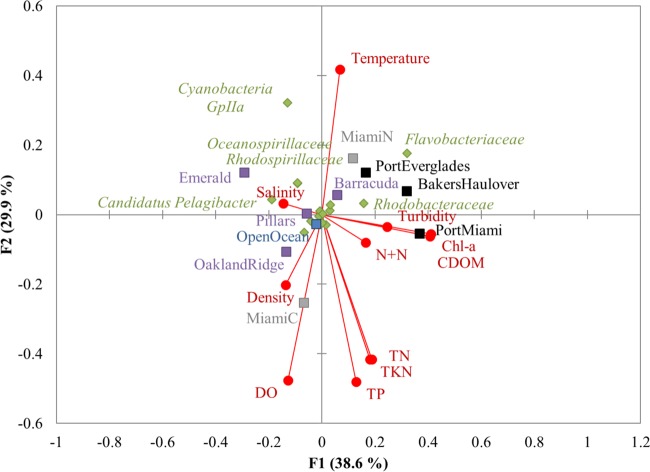
Redundancy analysis relating physicochemical parameters, sample site locations, and distributions of the 15 most abundant bacterial families among water samples. Families that clustered around the origin are not shown for clarity. Colors denote sample type: purple, reef water; blue, open ocean; black, inlets; gray, outfalls. Chl-a, chlorophyll *a*.

Physicochemical parameters were presumed to vary primarily as a result of inputs from LBSP associated with changes between wet and dry seasons (30), with the exception of temperature. In order to determine which physicochemical factors and sampling locations best explained the variation in phylogenetic structure, RDA relating sampling site (independent variables, *n* = 10), physiochemical parameters (independent variables, *n* = 11), and the relative abundance of bacterial families (dependent variable, *n* = 15) among water samples was conducted (Fig. 4). The abundances of only a few prokaryotic families were related to physicochemical parameters, while the majority clustered near the origin. Based on the position in the same quadrant of the RDA, salinity appeared to be positively related with abundances of “Candidatus Pelagibacter,” Oceanospirillaceae, and Rhodospirillaceae and negatively associated with Flavobacteriaceae and Rhodobacteraceae. The abundance of Cyanobacteria group II was also positively associated with water temperature, based on similar direction and positioning in the RDA. Similar to the analysis of variance (ANOVA) results (described above), the Miami Central outfall was associated with increased nutrient concentrations, water density, and dissolved oxygen, while inlet samples were associated with higher concentrations of nitrate plus nitrite N, chlorophyll *a*, turbidity, and colored dissolved organic matter (CDOM).

Binary logistic regression analysis revealed that prokaryotic community composition, determined using abundant families, was not significantly related to HPyV detection (*P* ≥ 0.998), as determined previously (29). However, the abundances of Bacillaceae and Methanomassiliicoccaceae were significantly positively correlated with the concentration of PMMoV (*r* = 0.366 and 0.413, *P* = 0.015 and 0.006, respectively). In contrast, abundances of Flavobacteriaceae, Rhodospirillaceae, Rhodobacteraceae, and Micromonasporaceae were negatively correlated with PMMoV concentrations (*r* = −0.335 to −0.708, *P* ≤ 0.027).

### Source contributions.

**FIG 5:**
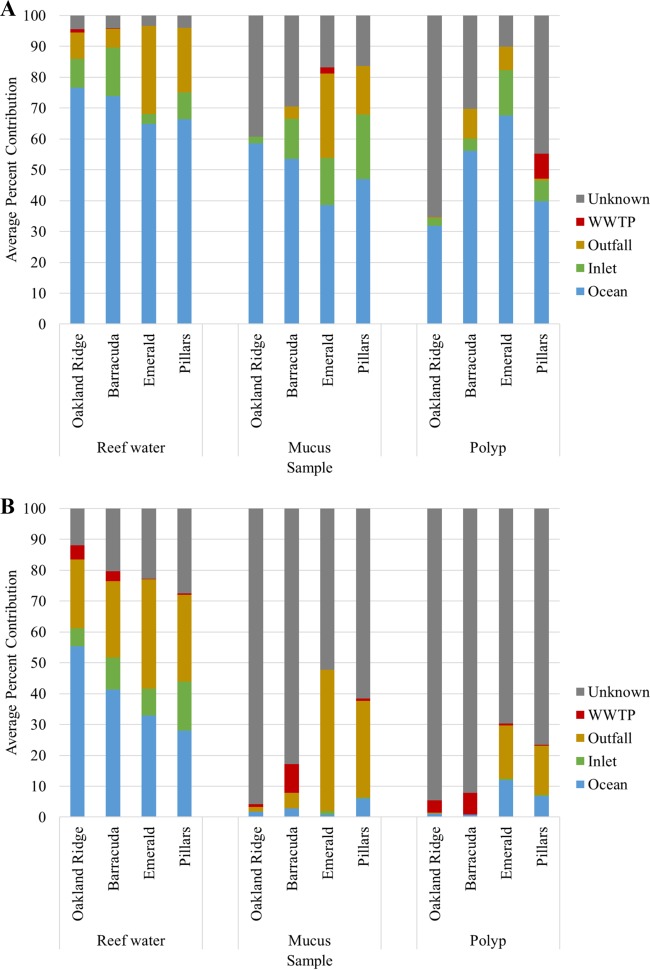
Percent prokaryotic community composition (A) and fungal community composition (B) attributable to source communities, as determined by SourceTracker.

Evaluation of prokaryotic source contributions using the SourceTracker sequence analysis software revealed that communities were predominantly composed of bacteria ubiquitous in marine samples, as well as host-specific OTUs among coral tissue samples (Fig. 5A). Among the LBSPs, outfall communities had the greatest influence on the designated sources on community composition among reef water and mucus samples, followed by the influence of communities from inlet samples. Among reef water samples, the contribution from outfall communities was significantly greater at sites collected from the southern region than others (Emerald and Pillars reefs, *P* = 0.007), although other source contributions did not differ significantly by site (*P* ≥ 0.052). The contribution of bacteria from source communities to those of reef water and polyps also showed temporal variability (Table S2) and were generally greater in samples collected in 2014 than those in 2015. Mucus communities did not show temporal variability in source influence (*P* ≥ 0.553, Table S2).

Fungal communities showed a lower proportion of ubiquitous marine taxa (open ocean water source) than did bacterial communities (Fig. 5B). The outfall contribution was predominant among reef water samples, with some influence from inlet communities, while coral mucus and polyp samples were predominantly composed of host-specific OTUs. Reef water samples collected in the northern region (Oakland Ridge and Barracuda reefs) had significantly higher percentages of the fungal communities associated with those from open ocean samples (*P* = 0.012), while reef communities in the southern region had significantly greater proportions of the community from unknown sources (*P* = 0.004). Similar to bacterial communities, no differences in source contributions by site were observed among mucus samples (*P* ≥ 0.083), although polyp communities differed significantly by site for all source categories (*P* ≤ 0.046), except that from WWTP (*P* = 0.114). Fungal source contributions showed less temporal variation than did those from prokaryotic communities (Table S3). Fungal contributions from inlets to reef water communities were greater in 2014, while outfall contributions to reef communities tended to be greater from May to November in both 2014 and 2015. Fungal contributions from outfall communities to polyp communities also tended to be higher in 2014.

## DISCUSSION

The prokaryotic communities characterized in this study using Illumina-based sequence technology were similar to those previously characterized in marine coastal waters in this area using 454 pyrosequencing technology (28). The marine water communities were primarily composed of Proteobacteria and Cyanobacteria, and members of the families Flavobacteriaceae and Rhodobacteraceae were found to vary in abundance between inlet and ocean/reef samples due, in part, to changes in salinity.

Communities from coral tissue samples showed some variation from previous reports (4, 5, 12). While Alphaproteobacteria, Gammaproteobacteria, and Cyanobacteria were the most abundant phyla, members of the Bacteroidetes and Firmicutes, previously reported to be associated with Porites astreoides (12), were detected here at low abundances. Instead, members of the class Bacilli, including the genera Bacillus and Paenibacillus, were abundant. Furthermore, the genus Endozoicomonas, previously reported to be an important member of a core coral microbiome (4), was abundant in samples at only at the Oakland Ridge reef, lying farthest north in the sampling area. Differences in taxonomic composition among studies may be due to the characterization method, including differences in primers used for next-generation sequencing (31), as well as geographic and physicochemical differences affecting coral communities (13).

Fungal communities in open ocean environments are only beginning to receive the same attention as prokaryotes and remain largely uncharacterized (32). While the majority of fungal community sequences were unclassified in this study, a previous report found members of class Sordariomycetes to be abundant in Porites astreoides near Panama (5). Among the genera classified, Bullera has recently been reported from marine seafloor sediment (33), but much less is known about the genus Dictyocatenulata, a stilbellaceous fungal group associated with bark and wood (34), which was among the only genus classified among coral samples. The fungal results, especially taxonomic classifications, presented here should be considered cautiously due to a number of issues that have recently received attention regarding the use of next-generation sequencing for ecological studies of fungi, including incomplete taxonomic databases and lack of a control mock community, sequencing target, and alignment and clustering methods (35, 36). Nevertheless, OTU-based analyses tended to agree well with those from prokaryotic community characterization (discussed below), suggesting that unclassified sequences may simply represent unexplored marine fungal diversity (32). Further analyses must await new sequencing technologies, bioinformatics approaches, and further development of taxonomic databases. Culture-based approaches may also benefit these analyses.

The prokaryotic and fungal communities were shown to differ significantly as a result of sample type (i.e., ocean, reef water, WWTP outfalls, and inlets). A previous study also characterized prokaryotic communities from ocean, reef, outfall, and inlet samples off the southeastern Florida coast and similarly found significant differences in the phylogenetic structures of communities associated with different sample types (28). This prior study, done using 454 technology, also noted that differences in community composition were associated with seasonal changes in rainfall (28). Seasonal variation of bacterial communities in coastal marine waters has been well established using next-generation sequencing approaches, with shifts in community structure associated with temperature, daylight, rainfall, and nutrient concentrations (28, 37). While seasonal changes in rainfall likely effect the magnitude of inputs from LBSP (30), this variation was presumed to affect sampling sites similarly in the current study.

Importantly, the differences in prokaryotic communities among types of water samples varied based on the area of the study region sampled. In the northern area, no differences were observed between outfall and ocean communities, suggesting minimal impacts from treated wastewater. However, this was not the case in the southern region, where the outfall communities differed significantly from those in both ocean and reef samples. Similarly, coral mucus communities could not be significantly differentiated from inlet communities in the southern region but were distinct from those of water samples in the northern area. Analyses of fungi generally agreed with prokaryotic results, although outfall samples differed from ocean samples in the northern area, and reef fungal communities were not significantly different from fungal communities among samples collected in the southern area. Taken together, these results suggest that there is a greater amount of anthropogenic input from both treated wastewater and LBSP from inlets into the southern area, as well as potentially different distribution dynamics between bacterial and fungal communities.

SourceTracker analysis (38) revealed that outfall communities had a greater influence on those in reef water than did inlet communities among both among prokaryotes and fungi. This result is not surprising given the greater similarity in physicochemical parameters between reef and outfall samples versus reef and inlet chemistries. It should also be noted that differences in source contribution may also be affected by salinity, temperature, oxygen, and nutrient gradients, which have previously been shown to influence the composition of both prokaryotic and fungal communities (32, 39, 40). Therefore, influence from outfall communities may possibly be exaggerated to some extent due to species sorting dynamics rather than community exchange. Not surprisingly, communities from coral mucus showed greater susceptibility to source influence than did the polyp communities, most likely due to a more intimate association of the coral mucus with the surrounding waters (41). Among prokaryotic communities, those of coral polyps at the Pillars reef site showed the greatest influence from WWTP, and both sites sampled in the southern region (i.e., Emerald and Pillars Reefs) showed higher fungal contributions from outfall. Taken together with the overall prokaryotic community analyses, these data suggest that anthropogenic sources have a greater impact on coral communities at the Emerald and Pillars reef sites than at Oakland Ridge and Barracuda reefs. Furthermore, viral microbial source tracking marker concentrations were more frequently detected (with HPyV) and found at higher concentrations (with PMMoV) from Miami Central outfall than from Miami North, suggesting a stronger influence of human fecal contamination in the southern sampling area.

In this study, it was found that microbial communities primarily from WWTP outfalls were the predominant influence from LBSP sources on communities in reef water and coral tissues. Furthermore, temporal variation was observed in source contributions, as expected based on seasonal dynamics (30), but geographic variation was also observed and corresponded to previously reported differences in human microbial source tracking markers (29). Previous studies have shown that the WWTP discharge plumes from these oceanic outfalls tend to rise to the surface, where they dilute out within a few kilometers of the outfalls and typically do not descend to the reefs themselves (42). However, an examination of the microbial community exchanges presented here suggests that microbial contaminants can and do reach the actual reef corals and may influence the community structure of reef microbiota (and thus presumably influence the health status and resiliency of reef ecosystems). While runoff contamination from LBSP has been well characterized (18), this study highlights the importance of the contribution of WWTP outfalls to reef contamination.

As corals face increasing environmental stressors in an era of climate change, the reduction of individual stressors that can be mediated, such as LBSP exposures, becomes ever more critical. Future research will be necessary to better inform how these variations in coral and coastal water microbial community structure influence the progression of diseases in order to better protect these dynamic ecosystems.

## MATERIALS AND METHODS

### Sample collection.

**FIG 6:**
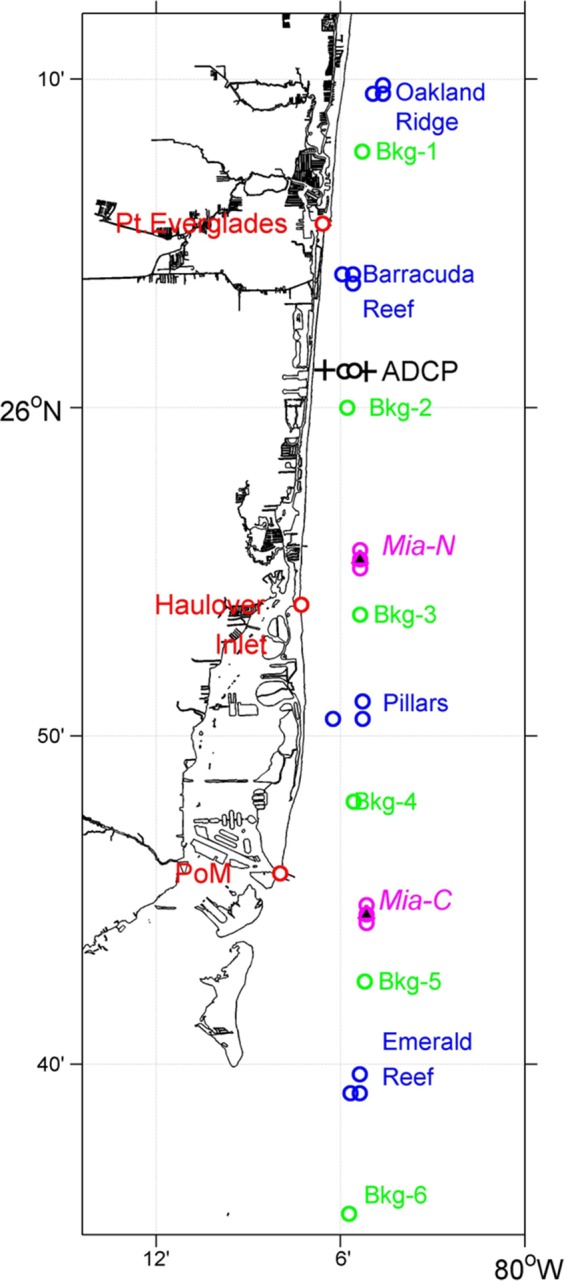
Map of sites sampled. Green, ocean; blue, reef; pink, outfall; red, inlet. Base map created with ArcMap (ArcGIS, version 9.1); shape files used were downloaded from NOAA's National Geodetic Survey website (https://www.ngs.noaa.gov/NSDE/).

Water and coral samples were collected during 2014 and 2015 from three coastal inlets (Port of Miami, Baker's Haulover, and Port Everglades), two treated wastewater effluents (Miami Central and Miami North), two surface boil expressions of oceanic outfalls from treated wastewater (Miami Central and Miami North), 16 coral reef water sites (four reefs with three sites each for surface water and four reefs with one site each for bottom), 12 coral polyp tissue extracts (four reefs with three sites each), and 12 coral mucus extracts (Fig. 6; described in detail in supplemental methods). Water samples were collected on 12 bimonthly 2-day sampling cruises as part of the FACE program Numeric Nutrient Criteria (NNC) cruises aboard the 41-foot National Oceanic Atmospheric Administration (NOAA) research vessel Hildebrand (described in detail in the supplemental methods). Coral tissue (polyps) and mucus samples were collected on a quarterly basis from 2014 to 2015 from two different coral species (Porites asteroids and Siderastrea siderea) from the same three reef sites at each of the same four sentinel reefs (Emerald, Pillars, Barracuda, and Oakland), described in detail in the supplemental methods.

### Sample processing and DNA extraction.

Water samples were processed and extracted as previously described (28). In brief, 1-liter water samples were aseptically filtered through 0.2-μm-pore-size 47-mm-diameter sterile mixed cellulose ester membrane filters (EMD Millipore, Billerica, MA, USA). Filters were aseptically transferred with sterile forceps to Lysing Matrix “A” bead-beating tubes (MP Biomedicals, LLC, Santa Ana, CA, USA), and stored frozen at −80°C until DNA extraction. DNA was extracted from filters using the FastDNA spin kit (MP Biomedicals), as per the manufacturer's directions, and stored frozen at −80°C until further analysis.

Coral mucus samples (60 ml) were aseptically filtered onto polycarbonate membrane filters (0.2-μm pore size, 47 mm diameter), aseptically transferred to Lysing Matrix “E” tubes (MP Biomedicals), and then extracted with the FastDNA spin kit for soil (MP Biomedicals), using the Lysing Matrix “E,” as per the manufacturer's directions. The coral polyp tissues were preserved in 95% ethanol and collected by aseptically filtering the material onto sterile polycarbonate membrane filters (0.2-μm pore size, 47 mm diameter). Large pieces of tissue were transferred from the filter into Lysing Matrix “E” tubes (MP Biomedicals) using sterile forceps. The filter was rolled using sterile forceps and aseptically placed into the same Lysing Matrix “E” tube with the rest of its tissue. DNA was then extracted using the FastDNA spin kit for soil (MP Biomedicals).

### Next-generation Illumina sequencing.

Prokaryotic sequencing was performed using the 515F/806R primer set targeting the V4 region (43), and fungal sequencing was performed using the ITS1F/ITS2 primer set targeting the internal transcribed spacer 1 (ITS1) region (44). Amplification and sequencing were performed using the dual-index method by the University of Minnesota Genomics Center (Minneapolis, MN, USA) (45), and each sample plate included a sterile-water negative control that was carried through amplification and sequencing. Sequencing was performed on the Illumina HiSeq 2500 and MiSeq platforms, and the results have been shown to be comparable across platforms (43).

### Bioinformatics.

All sequence processing, unless otherwise noted, was performed using mothur software version 1.34.0 (46). Prokaryotic sequence data were trimmed to the first 160 nucleotides (nt) and paired-end joined using the fastq-join software (25). Sequences were trimmed for quality, as described previously for V5 and V6 data (26). Global alignment was performed against the SILVA database version 119 (27), sequences were subjected to a 2% preclustering step to remove sequence errors (47), and chimeric sequences were identified and removed using UCHIME (48). Operational taxonomic units were assigned at ≥97% identity by complete-linkage clustering. Taxonomic assignments were made against Ribosomal Database Project version 14 (49) at a bootstrap cutoff of 60%, as described previously (50). Fungal sequence data were trimmed to the first 150 nt and processed in the same way as the prokaryotic data, with the exception that sequences with homopolymers of >9 nt were removed, and fungal assignments were made using the UNITE database version 6 (51). For statistical comparisons, the prokaryotic data set was rarefied by random subsample to 35,000 sequence reads per sample, prior to OTU calling, and the fungal data set was rarefied to 10,000 reads per sample (52).

To evaluate potential contributions from LBSP, the software SourceTracker version 0.9.8 was used to analyze the sequencing data, with the default parameters (38). This software utilizes a Bayesian algorithm to identify OTUs from source communities found in sink communities at rarefaction to 1,000 sequence reads. The microbial communities from (i) treated wastewater effluent before oceanic discharge, (ii) treated wastewater oceanic outfalls (at the surface), (iii) coastal inlet discharge waters, and (iv) open ocean surface water communities were designated the sources for analysis by this SourceTracker algorithm. The open ocean background communities were included as sources to reduce noise associated with outfall samples, due to high community similarity between these sample types.

### Statistical analysis.

Since samples were collected on adjacent sampling dates, representing a northern sampling area and a southern sampling area, analyses were performed separately with respect to sampling region to take into account temporal and geographic differences among samples. Statistics were calculated using mothur, unless otherwise stated. The Shannon index and abundance-based coverage estimate (ACE) parameters were calculated as parametric and nonparametric measures of diversity, respectively. Differences in beta diversity were evaluated using analysis of similarity (ANOSIM) (53) based on Bray-Curtis dissimilarity distances (54). Nonparametric differences in OTU abundances were evaluated using the Kruskal-Wallis test (55). Ordination of Bray-Curtis dissimilarities was performed using principal-coordinate analysis (PCoA) (56), and significance of clustering was evaluated using analysis of molecular variance (AMOVA) (57). ANOVA with Tukey's *post hoc* test, Spearman correlation, binary logistic regression, and redundancy analyses were performed using XLSTAT software version 2015.1.01 (Addinsoft, Belmont, MA, USA). All statistics were evaluated at an α value of 0.05. For redundancy analysis (RDA), the 15 most abundant families were included in the analysis, normalized as percentage of total sequence reads per sample. Physicochemical variables were transformed to a number between 0 and 1 in XLSTAT for RDA.

### Accession number(s).

The sequence data are deposited in the Sequence Read Archive of the National Center for Biotechnology Information under BioProject accession number SRP076111.

## Supplementary Material

Supplemental material
