# Supplementary material for: Differential Impacts of Land-Based Sources of Pollution on the Microbiota of Southeast Florida Coral Reefs
Source: Appl Environ Microbiol. 2017 May 1;83(10):e03378-16. doi: 10.1128/AEM.03378-16 (PMC5411493; doi:10.1128/AEM.03378-16)
Supplement: Supplemental material [file supp_83_10_e03378-16__index.html]

Supplemental material 

# Differential Impacts of Land-Based Sources of Pollution on the Microbiota of Southeast Florida Coral Reefs

## Supplemental material

- Supplemental file 1 -

  Supplemental methods: sampling location, sample collection; supplemental results: fungal community diversity and composition; detection and quantification of PMMoV and HPyV (Table S1); percentages of prokaryotic (Table S2) and fungal (Table S3) source community contributions to reef, mucus, and polyp sinks; distribution of abundant genera in coral tissue samples (Fig. S1); family-level classification of OTUs that varied significantly among sample types by Kruskal-Wallis test (*P* < 0.05) during sampling days 1 and 2 (Fig. S2); redundancy analysis relating abundances of bacterial families to sample sites and types (Fig. S3).

  PDF, 470K
