# Supplementary material for: Differential Impacts of Land-Based Sources of Pollution on the Microbiota of Southeast Florida Coral Reefs
Source: Appl Environ Microbiol. 2017 May 1;83(10):e03378-16. doi: 10.1128/AEM.03378-16 (PMC5411493; doi:10.1128/AEM.03378-16)
Supplement: Supplemental material [file AEM.03378-16_zam999117826s1.pdf]

SUPPLEMENTARY MATERIALS

A Next-Generation Sequencing Approach to Characterize the Impacts of Land-based Sources of Pollution on the Microbiota of Southeast Florida Coral Reefs

Christopher Staley[1], Thomas Kaiser[1], Maribeth L. Gidley[2,3], Ian C. Enochs[2,3], Paul R. Jones[2,3], Kelly D. Goodwin[2], Christopher D. Sinigalliano[2], Michael J. Sadowsky[1†], and Chan Lan Chun[4,†,*]

[1]BioTechnology Institute, University of Minnesota, St. Paul, Minnesota, USA; [2]Atlantic Oceanographic and Meteorological Laboratory, National Oceanographic and Atmospheric Administration, Miami, Florida, USA; [3]Cooperative Institute for Marine and Atmospheric Studies, University of Miami, Miami, Florida, USA; [4]Department of Civil Engineering and National Resources Research Institute, University of Minnesota Duluth, Duluth, Minnesota, USA

[*]Corresponding Author: Chan Lan Chun, Department of Civil Engineering and National Resources Research Institute, University of Minnesota Duluth, Duluth, Minnesota, USA; Swenson Civil Engineering 221, 1405 University Drive, Duluth, MN 55812; Phone: (218)-788-2613, Email: chun0157@d.umn.edu [†]These authors contributed equally.

Supplementary Methods

Sampling location

The Southeast Florida coast, specifically the counties of Miami-Dade and Broward (~25.2 to 26.3°N latitude), contain some 126 km of coastline extending approximately parallel to the flow of the Florida Current to the east. The region contains three non-continuous reef tracts (1) containing ecosystems of significant economic, ecological and aesthetic value (2, 3) to the 4.2 million residents therein (as of the 2010 US Census, [http://quickfacts.census.gov/qfd/states/12000.html]). This area also contains significant point sources of pollution, i.e., coastal ocean inlets and ocean effluent outfalls of three municipal wastewater treatment plants (WWTP) (4). The region is drained by an extensive system of drainage canals which have significant impact on the surface and ground water flow (5) and remain a major conduit for agricultural and urban runoff (6). These may contain chemical fertilizers, pesticides, suspended solids and highly colored organic materials, elevated nutrients, high natural iron concentrations, and contaminants from septic tanks and landfills (3). In south Broward and north Miami-Dade, the canal waters are conducted to the coastal ocean through three inlets: Port Everglades, Baker's Haulover, and the Port of Miami.

Sample collection

The Water Quality field program incorporated two years of 12 bimonthly 2-day water quality cruises from Port Everglades to Miami and return, with the southern region sites south of Haulover Inlet collected on Day 1 of each cruise and the northern region sites north of Haulover inlet collected on Day 2 of each cruise. Water samples and *in situ* sensor data were collected with

a Seabird ECO 55 rosette sampler holding six 4-L seawater sampling Niskin-type bottles and a SBE 19V2 CTD (conductivity-temperature-density) instrument package, sampling at 4 Hz. The CTD and rosette assembly ("CTD") was lowered into the water column at the specified sampling locations with all bottles open to seawater flow; two bottles were closed off at the bottom, and mid-depth, and near the surface to capture the water at those depths. Water sub-samples were collected from the Niskin bottle discharge ports into sterile 2 liter polypropylene bottles and held on ice for return to the lab and sample processing.

Coral tissue and mucus samples were collected on a quarterly basis from 2014-2015 aboard the NOAA small boat *R/V Cable* during the Numeric Nutrient Criteria Study (NNCS) coral benthic surveys. Coral tissue and mucus samples were collected from two different coral species (*Porites asteroids* and *Siderastrea siderea*) from the same three reef sites at each of the same four sentinel reefs (Emerald, Pillars, Barracuda, and Oakland). Coral tissue was collected by syringe biopsy, where the syringe was held firmly against the coral skeleton and the plunger pulled back, causing a suction that draws the entire coral polyp into the syringe (while causing no damage to the coral skeleton – and the coral tissue typically grows back within several weeks). Two replicate clonal polyps were collected from the same coral head for each particular coral tissue sample in a sterile 5mL syringe by SCUBA divers conducting the coral benthic survey. Upon surfacing, coral tissue from the syringe was transferred to a sterile 50 mL polypropylene tube and filled with absolute ethanol for a final preservative concentration of 95% ethanol (EtOH). Ethanol-preserved coral tissue samples were held on ice until return to the lab for processing, Coral mucus was collected by the benthic survey divers from the same coral heads, using 60 mL syringes, gently sucking near the coral surface but not strongly enough to dislodge coral tissue. Upon return to boat, mucus samples were also held on ice until return to

the lab for processing. Mucus samples were filtered (see below) immediately upon return to the lab. Coral polyp tissues in 95% EtOH were stored in freezer at -20°C, until DNA extraction (see below).

Supplementary Results

Fungal community diversity and composition

Alpha diversity, measured by both Shannon and ACE indices (Table 3), was significantly lower in coral compared to other water and WWTP samples ($P < 0.0001$ for both indices). In contrast to prokaryotic results in which open ocean samples showed the highest alpha diversity, the WWTP samples had significantly higher fungal alpha diversity, by both indices, than did water samples, which generally did not vary significantly from each other. Differences in Shannon indices among sites were not significantly different among samples of the same type, which was similarly observed among prokaryotic communities. However, differences in ACE richness were significant among inlet and reef water samples, where Port Everglades had significantly higher richness than Baker's Haulover ($P = 0.023$) and the Pillars reef site had significantly greater richness than did the Oakland Ridge site ($P = 0.008$).

Differences in beta diversity followed similar trends to those observed for prokaryotic communities. Among samples from the northern region, fungal communities from inlet and outfall samples did not differ significantly ($P = 0.722$), and reef and open ocean communities were also similar ($P = 0.688$), as evaluated by ANOSIM. Inlet/outfall sample communities differed significantly from reef/ocean samples ($P \leq 0.019$). While the fungal community from outfall sites did not differ significantly from those of the WWTP samples ($P = 0.187$), all other

water samples had significant differences in beta diversity relative to the WWTP samples ($P \leq$ 0.026). In contrast, no significant differences in community composition were observed among ocean, reef, and inlet samples ($P \geq 0.049$) in the southern region, which had significantly different composition than outfall samples ($P \leq 0.036$). Outfall samples, however, had significantly different composition than WWTP samples ($P = 0.001$).

Table S1. Detection and quantification of PMMoV and HPyV Virsues.

Site	Virus Type			
	PMMoV		HPyV	
	n	Concentration (gene copies L^{-1})	n	% positive
Miami – Central outfall*	18	$2.31 \pm 3.06 \times 10^4$	18	66.7
Miami – North outfall	18	$1.66 \pm 4.19 \times 10^4$	18	38.9
Port Everglades inlet	6	$1.86 \pm 2.52 \times 10^2$	6	33.3
Bakers Haulover inlet	6	$1.85 \pm 2.13 \times 10^2$	4	25.0
Port Miami inlet	6	$4.06 \pm 7.55 \times 10^2$	6	16.7

*Outfall samples include all samples collected within 1 km of the outfall.

Table S2 – Percent of prokaryotic source community contribution to reef, mucus, and polyp sinks. A) Inlet, B) outfall, and C) WWTP source contributions.

A)

Month	Reef	Mucus	Polyp
Nov '13	1.9 ± 4.2[*]	NA[†]	NA
Jan '14	0.4 ± 1.5	NA	NA
Mar '14	2.3 ± 8.2	NA	NA
May '14	18.3 ± 35.8	NA	NA
Jun '14	NA	15.6 ± 25.1	16.5 ± 13.6[a,b]
Jul '14	24.6 ± 31.9	NA	NA
Sep '14	19.4 ± 27.2	14.3 ± 12.2	27.6 ± 30.3[b]
Nov '14	18.4 ± 29.3	NA	NA
Dec '14	NA	13.7 ± 21.6	23.3 ± 32.5[a]
Jan '15	4.3 ± 11.0	NA	NA
Mar '15	2.3 ± 4.8	NA	0.0 ± 0.0[b]
May '15	0.9 ± 2.2	NA	NA
Jun '15	NA	NA	0.8 ± 38.5[b]
Jul '15	0.1 ± 0.1	NA	NA
Sep '15	NA	NA	0.0 ± 0.0[b]
P-value[‡]	0.002	0.982	< 0.0001

B)

Month	Reef	Mucus	Polyp
Nov '13	1.3 ± 1.6	NA	NA
Jan '14	1.4 ± 1.5	NA	NA
Mar '14	0.7 ± 1.5	NA	NA
May '14	24.1 ± 41.1	NA	NA
Jun '14	NA	9.2 ± 17.3	27.4 ± 42.8[a]
Jul '14	31.4 ± 40.0	NA	NA
Sep '14	28.5 ± 38.6	13.9 ± 25.5	12.9 ± 14.6[a,b]
Nov '14	32.6 ± 39.7	NA	NA
Dec '14	NA	13.9 ± 24.7	7.1 ± 11.9[a,b]
Jan '15	32.0 ± 43.1	NA	NA
Mar '15	0.7 ± 0.9	NA	0.0 ± 0.0[b]
May '15	2.1 ± 2.1	NA	NA
Jun '15	NA	NA	0.0 ± 0.0[b]
Jul '15	0.1 ± 0.2	NA	NA
Sep '15	NA	NA	0.1 ± 0.2[b]
P-value[‡]	< 0.0001	0.892	0.002

C)

Month	Reef	Mucus	Polyp
Nov '13	0 ± 0	NA	NA
Jan '14	0 ± 0	NA	NA
Mar '14	0 ± 0	NA	NA
May '14	0.9 ± 3.4	NA	NA
Jun '14	NA	0.0 ± 0.0	25.2 ± 39[a]
Jul '14	0.0 ± 0.0	NA	NA
Sep '14	1.6 ± 4.9	1 ± 3.5	0.0 ± 0.0[b]
Nov '14	0.5 ± 1.2	NA	NA
Dec '14	NA	0.0± 0.0	0.0 ± 0.0[b]
Jan '15	0.1 ± 0.1	NA	NA
Mar '15	0 ± 0	NA	0.0 ± 0.0[b]
May '15	0 ± 0	NA	NA
Jun '15	NA	NA	0.0 ± 0.0[b]
Jul '15	0.0 ± 0.0	NA	NA
Sep '15	NA	NA	0.0 ± 0.0[b]
P-value[‡]	0.358	0.553	0.001

[*]Vales are mean ± standard deviation of replicate samples.

[†]NA: not applicable.

[‡]ANOVA analyses were run on each column separately.

[a,b]Samples sharing the same superscript did not differ significantly by Tukey's *post-hoc* test ($P > 0.05$). Where the ANOVA result was significant ($P < 0.05$), but superscripts are not shown, no *post-hoc* differences were significant.

Table S3 – Percent of fungal source community contribution to reef, mucus, and polyp sinks. A) Inlet, B) outfall, and C) WWTP source contributions.

A)

Month	Reef	Mucus	Polyp
Nov '13	0.0 ± 0.0*	NA†	NA
Jan '14	0.0 ± 0.0	NA	NA
Mar '14	0.5 ± 1.4	NA	NA
May '14	11.1 ± 25.1	NA	NA
Jun '14	NA	0.3 ± 0.9	0.6 ± 1.2
Jul '14	27.2 ± 36.8	NA	NA
Sep '14	16.9 ± 25.3	0.4 ± 1.1	0 ± 0
Nov '14	14.5 ± 20.0	NA	NA
Dec '14	NA	0.0 ± 0.0	0.0 ± 0.0
Jan '15	12.2 ± 17.7	NA	NA
Mar '15	0.6 ± 1.6	NA	0.2 ± 0.5
May '15	8.7 ± 14.2	NA	NA
Jun '15	NA	NA	0.2 ± 0.6
Jul '15	1.6 ± 3.7	NA	NA
Sep '15	NA	NA	0.2 ± 0.6
P-value‡	0.021	0.561	0.246

B)

Month	Reef	Mucus	Polyp
Nov '13	0.0 ± 0.0[a]	NA	NA
Jan '14	3.2 ± 5.6[a,b]	NA	NA
Mar '14	13.9 ± 14.8[a,b]	NA	NA
May '14	22.4 ± 37.9[a,b,c]	NA	NA
Jun '14	NA	10.4 ± 31.2	30.6 ± 45.9[a]
Jul '14	37.6 ± 42.8[a,b,c]	NA	NA
Sep '14	62.3 ± 28.4[c]	22.2 ± 40.5	17.1 ± 37.7[a,b]
Nov '14	36.0 ± 35.1[a,b,c]	NA	NA
Dec '14	NA	28.6 ± 42.1	15.2 ± 35.5[a,b]
Jan '15	43.0 ± 28.0[b,c]	NA	NA
Mar '15	12.1 ± 11.8[a,b]	NA	0.2 ± 0.8[b]
May '15	35.3 ± 17.5[a,b,c]	NA	NA
Jun '15	NA	NA	0.0 ± 0.0[b]
Jul '15	0.0 ± 10.2[a,b]	NA	NA
Sep '15	NA	NA	0.2 ± 0.6[b]
P-value‡	< 0.0001	0.610	0.007

C)

Month	Reef	Mucus	Polyp
Nov ‘13	0.0 ± 0.0	NA	NA
Jan ‘14	7.5 ± 18.1	NA	NA
Mar ‘14	0.0 ± 0.0	NA	NA
May ‘14	0.9 ± 2.8	NA	NA
Jun ‘14	NA	0.0 ± 0.0	0.5 ± 1.4
Jul ‘14	0.0 ± 0.0	NA	NA
Sep ‘14	0.1 ± 0.4	0.5 ± 1.3	0.6 ± 1.1
Nov ‘14	6.9 ± 14.6	NA	NA
Dec ‘14	NA	11.1 ± 29.4	0.7 ± 1.7
Jan ‘15	6.1 ± 13.7	NA	NA
Mar ‘15	0.0 ± 0.0	NA	7.9 ± 17.1
May ‘15	0.5 ± 1.7	NA	NA
Jun ‘15	NA	NA	0.0 ± 0.0
Jul ‘15	0.0 ± 0.0	NA	NA
Sep ‘15	NA	NA	4.4 ± 12.1
P-value[‡]	0.220	0.236	0.224

[*]Vales are mean ± standard deviation of replicate samples.

[†]NA: not applicable.

[‡]ANOVA analyses were run on each column separately.

[a,b]Samples sharing the same superscript did not differ significantly by Tukey’s post-hoc test (P > 0.05). Where the ANOVA result was significant (P < 0.05), but not superscripts are shown, no post-hoc differences were significant.

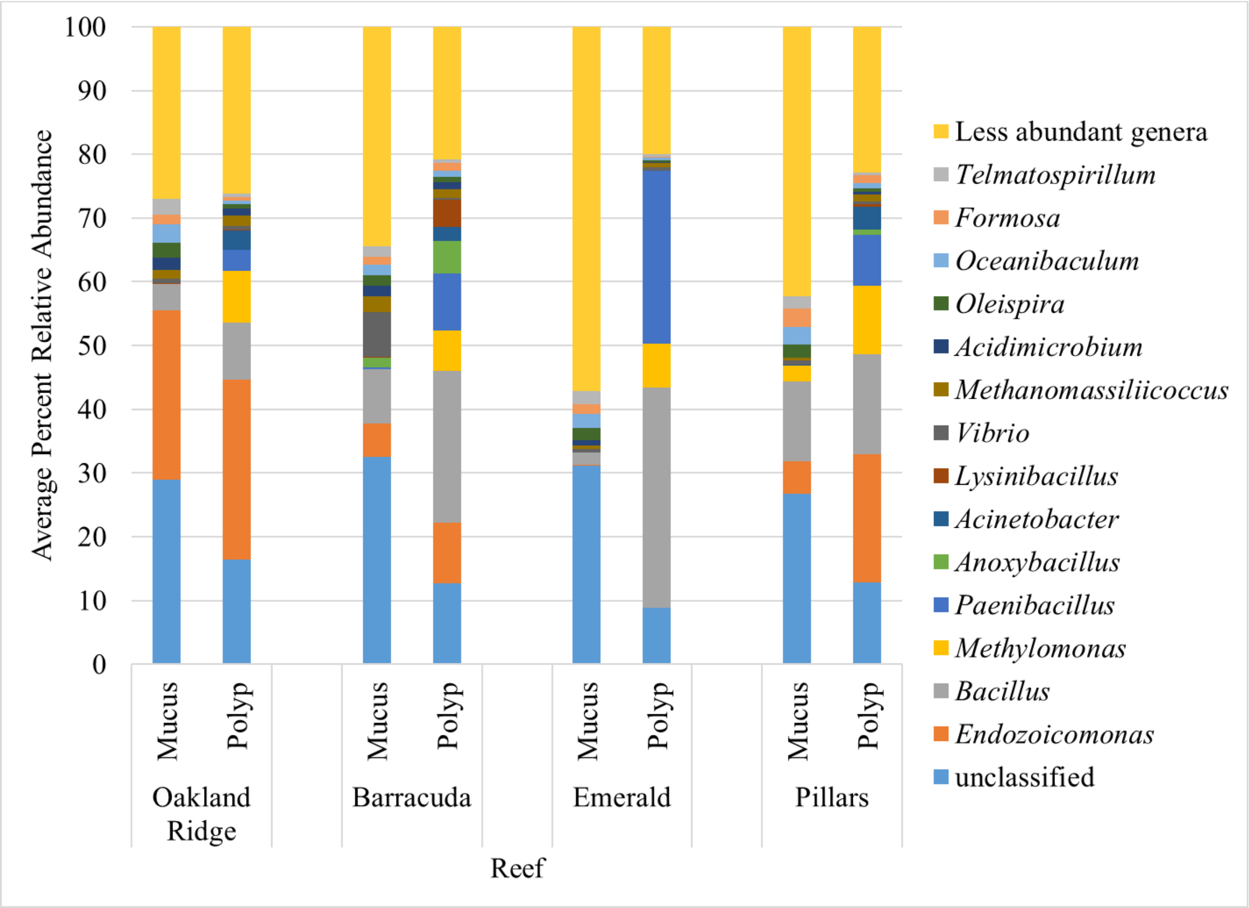

Figure S1. Distribution of abundant genera in coral tissue samples.

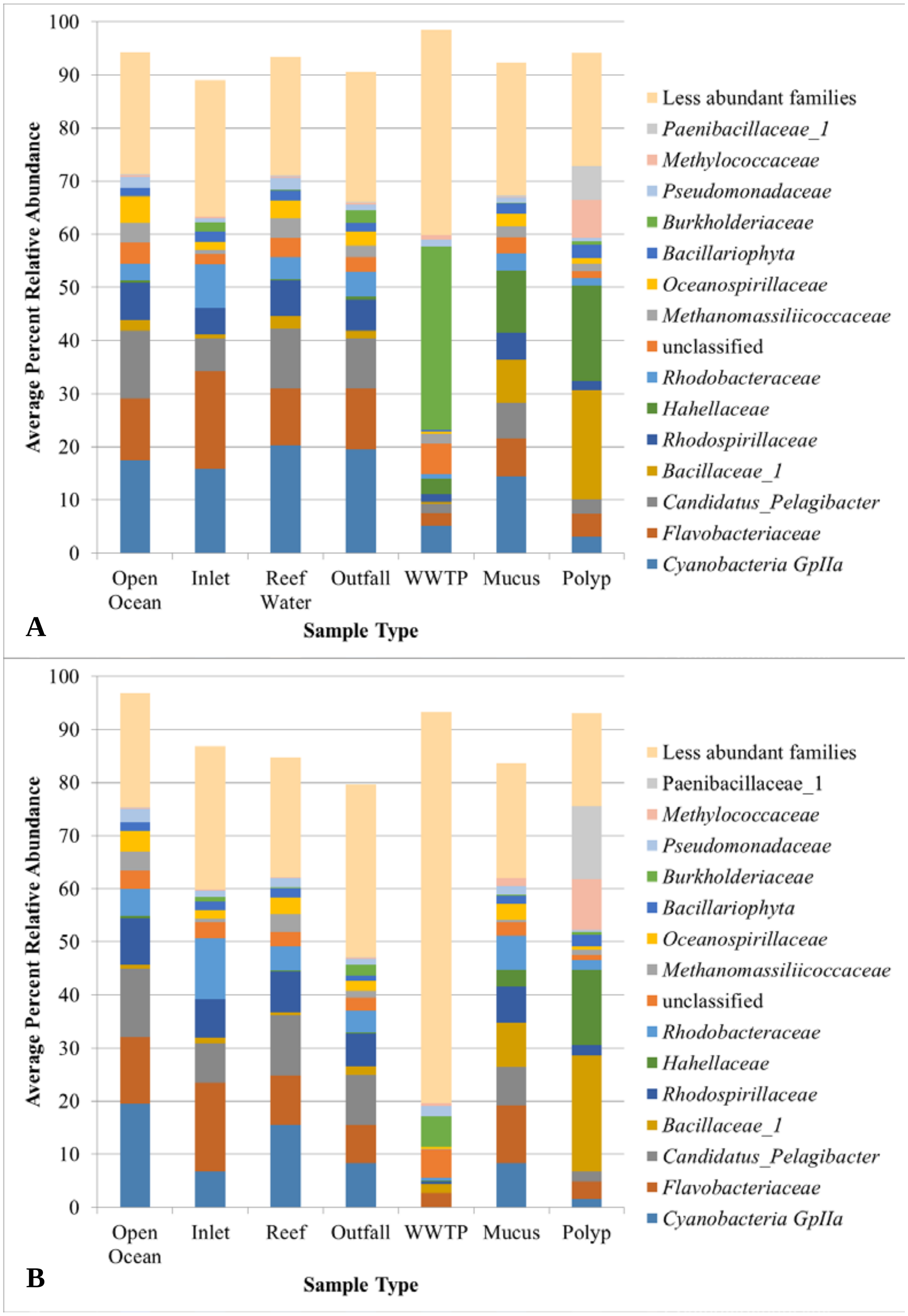

Figure S2. Family-level classification of OTUs that varied significantly among sample types by Kruskal-Wallis test ($P < 0.05$) during (A) sampling day 1 and (B) sampling day 2.

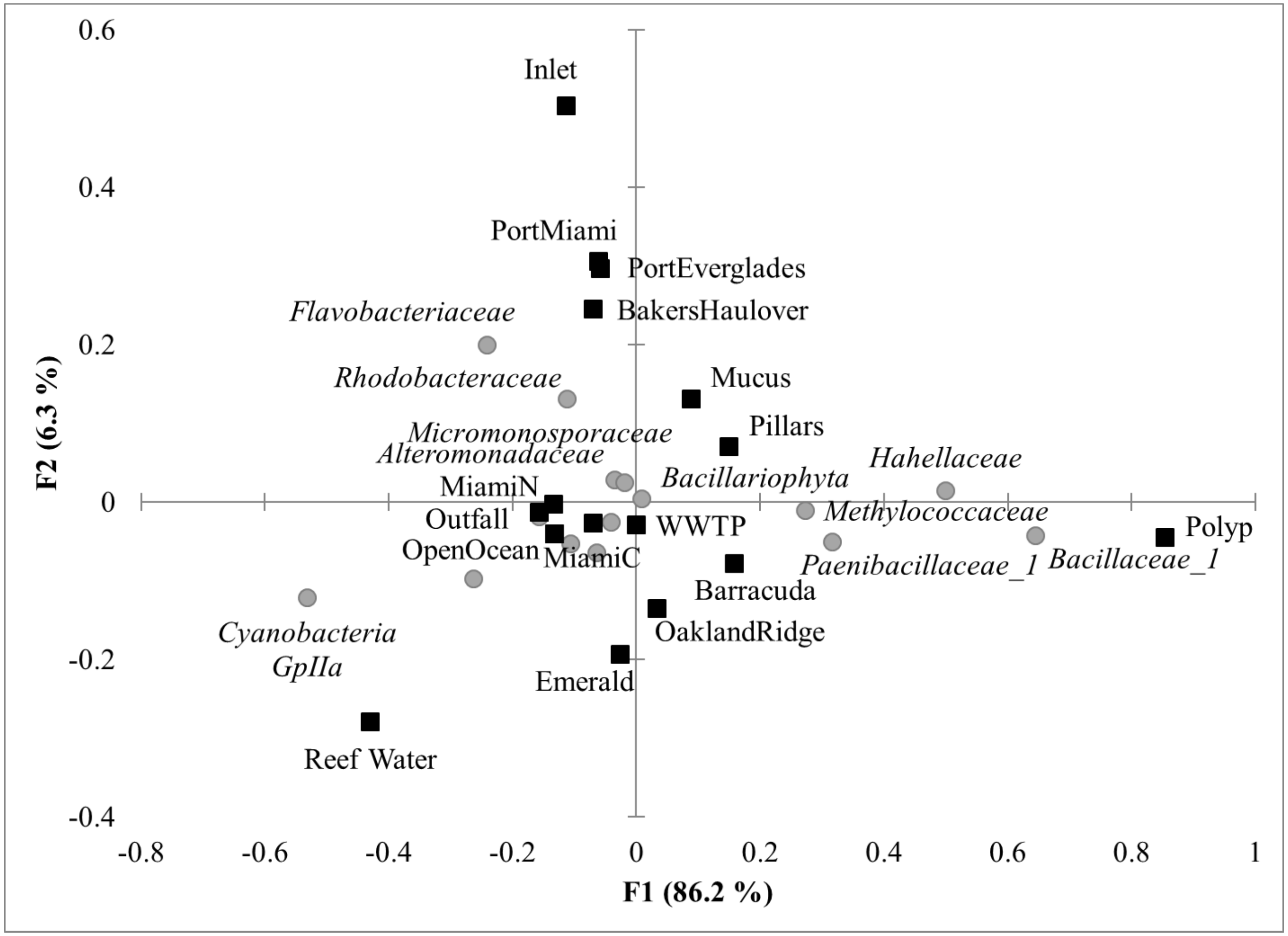

Figure S3. Redundancy analysis relating abundances of bacterial families to sample sites and types. Unlabeled families around the origin include *Candidatus Pelagibacter*, *Methanomassiliicoccaceae*, *Oceanospirillaceae*, *Pseudomonadaceae,* and *Rhodospirillaceae.* The 15 most abundant families were included in the analysis. Legend: square = sampling location, circle = prokaryotic family.

References

1. **Banks KW**, **Riegl BM**, **Richards VP**, **Walker BK**, **Helmle KP**, **Jordan LKB**, **Phipps J**, **Shivji MS**, **Spieler RE**, **Dodge RE**. 2008. The Reef Tract of Continental Southeast Florida (Miami-Dade, Broward and Palm Beach Counties, USA), p. 175–220. *In* Coral Reefs of the USA. Springer Netherlands, Dordrecht.

2. **Johns GM**, **Leeworthy VR**, **Bell FW**, **Bonn MA**. 2001. Socioeconomic study of reefs in southeast Florida.

3. **Gregg K**. 2013. Literature Review and Synthesis of Land-Based Sources of Pollution Affecting Essential Fish Habitats in Southeast Florida.

4. **Koopman B**, **Heaney JP**, **Cakir F**, **Rembold M**, **Indeglia P**, **Kini G**. 2006. Ocean Outfall Study Final Report.

5. **Sklar F**, **McVoy C**, **VanZee R**, **Gawlik D**, **Tarboton K**, **Rudnick D**, **Maiao S**, **Armentano T**. 2002. The Effects of Altered Hydrology on the ecology of the Everglades, p. 39–82. *In* Porter, J, Porter, K (eds.), The Everglades, Florida Bay, and Coral Reefs of the Florida Keys, An Ecosystem Sourcebook. CRC Press, New York.

6. **SWFMD**. 2010. Canals in South Florida: A Technical Support Document, Appendix A; Basic Concepts, Hydrologic Terminology, Glossary of Terms and Abbreviations. West Palm Beach, FL.
